# 3D Localization of Heat Sources Using LiDAR–Thermal Data Fusion and Multisensor Calibration

**DOI:** 10.3390/s26123876

**Published:** 2026-06-18

**Authors:** Rafał Gasz, Mateusz Pluskota, Krzysztof Schwierz

**Affiliations:** 1 Department of Artificial Intelligence, Faculty of Computer Science, Opole University of Technology, Prószkowska 76 St., 45-758 Opole, Poland; 2 KBA AUTOMATIC, Technologiczna 2A St., 45-839 Opole, Poland; m.pluskota@kba-automatic.pl (M.P.); k.schwierz@kba-automatic.pl (K.S.)

**Keywords:** LiDAR, thermal imaging, multisensor calibration, 3D thermal mapping, sensor fusion, temperature localization, thermal anomaly detection, point cloud

## Abstract

Integration of LiDAR and thermal sensing has become increasingly important in robotics, infrastructure diagnostics, environmental monitoring, and autonomous perception systems. LiDAR sensors provide accurate three-dimensional geometric information but do not directly capture thermal properties of observed objects, whereas thermal cameras provide temperature distributions without explicit spatial structure. Fusion of both sensing modalities enables thermally augmented 3D scene reconstruction and spatial localization of temperature anomalies. This paper presents a practical LiDAR–thermal fusion framework for three-dimensional localization of heat sources using an Ouster OS1 LiDAR sensor and a FLIR A70 thermal camera. The proposed framework includes intrinsic thermal-camera calibration, extrinsic LiDAR–thermal calibration, multimodal data synchronization, projection of LiDAR points onto the thermal image plane, and assignment of temperature values to spatial points. Additionally, a dedicated thermally distinguishable calibration target is proposed to enable reliable multimodal feature extraction under low-contrast LWIR imaging conditions. The developed framework was experimentally validated using real radiometric thermal data and LiDAR point clouds acquired under laboratory conditions. Quantitative evaluation demonstrated reprojection errors below 1 pixel and a mean hottest-point localisation error of approximately 4.1 cm at a distance of 12.3 m. The results confirm that accurate spatial localisation of thermal anomalies can be achieved using a geometry-based multimodal fusion approach without relying on computationally expensive learning-based methods. The proposed framework emphasises practical deployment, deterministic calibration, and applicability in scenarios where limited training data or constrained computational resources make learning-based approaches difficult to apply. The proposed system may be applied to building energy diagnostics, industrial inspection, technical infrastructure monitoring, and robotic perception systems that require reliable spatial localisation of heat sources under real measurement conditions.

## 1. Introduction

Environmental perception systems based on spatial data are currently a key research area in robotics, autonomous systems, and the analysis of technical infrastructure. A three-dimensional representation of a scene enables accurate reconstruction of the geometric structure of the environment, which is applied, among others, in mobile robotics, decision support systems, intelligent transportation systems, and building energy diagnostics [1,2,3]. In many applications, it is important not only to reconstruct the environment’s geometry but also to obtain additional information about the physical properties of observed objects.

One of the most commonly used sensors for spatial data acquisition is a LiDAR (Light Detection and Ranging) scanner, which enables the generation of accurate three-dimensional point clouds representing the structure of the scene [4,5]. LiDAR technology enables precise distance measurements independent of lighting conditions and provides high spatial resolution of the acquired data, making it particularly useful for applications requiring accurate reconstruction of environmental geometry [6]. However, LiDAR data provide only geometric information and limited radiometric information (e.g., return intensity), making it difficult to identify the physical properties of objects, such as surface temperature.

A complementary source of information about physical properties of objects are thermal cameras, which enable measurement of temperature distribution on the surfaces of observed elements of the scene. Thermography is used, among other applications, for building energy diagnostics, industrial infrastructure monitoring, and the detection of thermal anomalies [7,8]. Thermal images provide information not available in the visible spectrum; however, the resulting data are two-dimensional and do not directly capture the spatial structure of the scene.

Due to the limitations of individual sensors, increasing attention has been given to multi-sensor systems and data fusion methods, enabling integration of information obtained from different measurement sources [9,10]. Integration of LiDAR and image data allows obtaining a more complete representation of the environment, combining accurate scene geometry with additional physical information [11]. Of particular importance is the integration of LiDAR data with thermal imagery, which provides an extended spatial representation enriched with temperature information [12,13].

Despite growing interest in multi-sensor data fusion, integrating LiDAR and thermal data poses several technical challenges. The most important problems include sensor-to-sensor calibration, data synchronization, and correct mapping of thermal information into three-dimensional space [14,15]. Additional difficulties arise from differences in spatial resolution, fields of view, and characteristics of the generated data. In particular, thermal cameras often have lower spatial resolution and limited image contrast, which complicates the calibration process and accurate data alignment [13].

Various approaches to multi-sensor data integration have been proposed in the literature, including both geometric methods and machine-learning-based solutions [16,17]. In many cases, data integration is performed by transforming LiDAR point clouds into the camera reference frame and projecting spatial points onto the image plane [18]. Despite progress in this field, there is still a limited number of works focusing on the practical implementation of complete measurement systems enabling integration of thermal and spatial data in real measurement conditions.

In contrast to classical LiDAR–RGB systems, integration of LiDAR and thermal cameras introduces additional challenges related to low image contrast, limited thermal texture, lower spatial resolution, and difficulties in extracting reliable multimodal correspondences. Although numerous calibration approaches have been proposed for RGB cameras, relatively few studies focus on practical LiDAR–thermal fusion systems operating on real radiometric thermal data under real measurement conditions. Furthermore, many existing approaches rely on complex optimisation frameworks or deep-learning-based models requiring large training datasets and high computational resources.

Therefore, there remains a need for practical, interpretable, and experimentally validated LiDAR–thermal fusion frameworks capable of accurate spatial localisation of thermal anomalies using real measurement data.

This paper presents a method for fusing LiDAR and thermal data for three-dimensional environmental analysis. The proposed approach includes the development of a complete measurement system, the implementation of a multi-sensor calibration procedure to estimate the transformation between sensor coordinate systems, and an algorithm for mapping thermal information onto LiDAR point clouds and determining the spatial coordinates of the hottest point. The effectiveness of the proposed method has been experimentally validated using real measurement data.

The main contributions of this work are as follows:

- Development of a practical LiDAR–thermal fusion framework dedicated to long-range spatial localization of thermal anomalies using radiometric thermal data and 3D point clouds;
- Proposal of a thermally distinguishable calibration target enabling reliable correspondence extraction in both LiDAR and LWIR thermal modalities under low-visibility conditions;
- Implementation of a complete end-to-end calibration and thermal mapping pipeline using real synchronized measurements from an Ouster OS1 LiDAR and a FLIR A70 thermal camera;
- Experimental validation of the proposed system under real measurement conditions, achieving sub-pixel reprojection accuracy and mean hottest-point localization error of 4.1 cm at a distance of approximately 12.3 m;
- Analysis of the influence of calibration point number and spatial distribution on thermal mapping accuracy and localization stability.

Unlike many existing LiDAR–camera calibration approaches focused primarily on RGB imaging or targetless optimisation methods, the proposed framework is specifically designed for practical fusion of LiDAR data with radiometric thermal imagery in low-feature thermal environments. The proposed approach prioritises interpretability, practical deployment, and reliable thermal anomaly localisation using real measurement data instead of simulation-based evaluation.

Unlike existing studies focusing primarily on calibration accuracy or multimodal perception, the proposed framework addresses the complete workflow from thermal-LiDAR calibration to three-dimensional localization of thermal anomalies using real radiometric thermal data. Particular emphasis is placed on practical deployment and direct estimation of the spatial coordinates of the hottest point.

## 2. Related Work

Integration of data from multiple sensors is an important research direction in environmental perception systems, particularly for improving scene reconstruction accuracy and increasing the amount of information obtained from a single measurement. In recent years, increasing interest has been observed in multi-sensor calibration methods and data fusion techniques combining LiDAR with various types of cameras, including RGB, multispectral, and thermal cameras [14,19]. Particular attention has been given to integrating spatial and thermal data, enabling the generation of a three-dimensional representation of the environment enriched with temperature information [12,20].

### 2.1. LiDAR–Camera Calibration

Mutual calibration of a LiDAR scanner and a camera is a key stage in multi-sensor data integration, as it directly affects the accuracy of mapping image information into three-dimensional space. The goal of calibration is to estimate the geometric transformation between the coordinate systems of the sensors, enabling projection of spatial points onto the image plane.

In the literature, both approaches based on dedicated calibration targets and methods relying on scene feature analysis have been proposed. Classical solutions are based on calibration objects such as checkerboards or geometric patterns and on minimising reprojection error in order to estimate the geometric relationship between sensor coordinate systems [19,21]. These methods are characterised by high accuracy; however, they require controlled measurement conditions and proper preparation of the calibration scene.

Alternative approaches use geometric features of the scene, such as planes, edges, or corners, enabling calibration without the use of dedicated targets [22,23]. Such solutions are often referred to as targetless methods and allow calibration in real environmental conditions. In recent years, methods based on global optimization, probabilistic estimation, and machine learning techniques have also been proposed, enabling automatic adjustment of calibration parameters [24,25].

Despite numerous studies, most works focus on integration of LiDAR with RGB cameras. Calibration using thermal cameras remains less common due to lower image contrast and a smaller number of distinctive features that can be used in the matching process [26]. Differences in data characteristics between sensors make automatic matching of corresponding points more difficult and increase sensitivity of the calibration process to measurement errors.

Table 1 compares the proposed framework with representative LiDAR–camera and LiDAR–thermal integration methods reported in the literature. Unlike many existing calibration approaches focused primarily on RGB cameras, the proposed system is specifically designed for integration of LiDAR data with radiometric thermal imagery.

Recent deep-learning-based approaches often provide increased robustness and automation capabilities; however, they typically require large annotated datasets, substantial computational resources, and complex training procedures. In contrast, the proposed framework relies on a geometry-based calibration strategy that remains fully interpretable and computationally efficient while providing accurate thermal anomaly localization using real measurement data.

Furthermore, compared with targetless calibration methods, the proposed approach utilizes a dedicated thermally distinguishable calibration target specifically designed for LWIR imaging conditions. This simplifies correspondence identification and improves calibration reliability in scenarios where thermal texture is limited or visible-light information is unavailable.

### 2.2. LiDAR–Thermal Fusion

Integration of LiDAR data and thermal images is more challenging than fusion of LiDAR and RGB data, mainly due to differences in spatial resolution, field of view, and signal characteristics. LiDAR data describe scene geometry in the form of a 3D point cloud, while a thermal camera provides a two-dimensional temperature distribution of object surfaces.

Various approaches to integration of these data have been proposed in the literature, including geometric methods as well as machine-learning-based solutions. One of the most commonly used approaches is transformation of LiDAR point clouds into the camera reference frame followed by projection of spatial points onto the thermal image plane [12,27]. This provides assignment of temperature values to spatial points and creation of an extended representation of the environment.

Recently, multimodal methods using data from multiple sensors have also been proposed in order to improve scene perception in challenging environmental conditions such as limited visibility or changing lighting conditions [28,29]. Integration of thermal and spatial data is applied, among others, in mobile robotics, autonomous transportation systems, and technical infrastructure monitoring.

Recently, deep-learning-based multimodal fusion approaches have also been proposed for thermal–LiDAR integration and cross-modal perception tasks. Such methods often improve robustness under challenging environmental conditions; however, they typically require large annotated datasets, high computational resources, and extensive training procedures. In contrast, geometry-based calibration frameworks remain attractive in practical engineering applications due to their interpretability, deterministic behavior, and relatively low computational complexity.

Despite increasing research interest, most existing works focus on specific applications or rely on simulated data. Relatively few studies present a complete pipeline for integration of LiDAR and thermal data, including data acquisition, multi-sensor calibration, and experimental validation under real measurement conditions.

Recent years have also seen increasing interest in multimodal fusion frameworks combining LiDAR and thermal sensing modalities for environmental perception, infrastructure inspection, autonomous systems, and thermal anomaly detection. Several studies have investigated direct integration of thermal imagery and LiDAR point clouds for three-dimensional thermal mapping, radiometric scene reconstruction, and sensor calibration. Examples include thermal–LiDAR calibration based on multimodal human feature matching [18], thermal-enhanced point cloud fusion approaches [30], and unified calibration frameworks integrating LiDAR, RGB, and thermal cameras [31].

At the same time, deep-learning-based calibration and multimodal fusion methods have gained significant attention. Recent research has explored transformer-based architectures and multimodal learning techniques for automatic LiDAR–camera calibration and sensor alignment [32,33]. Such approaches often improve automation and robustness; however, they typically require large annotated datasets, substantial computational resources, and extensive training procedures.

Recent review studies have also highlighted the growing role of thermal sensing in multimodal perception systems and identified increasing interest in combining geometric and thermal information for monitoring, inspection, and autonomous operation tasks [34]. Despite recent progress, practical LiDAR–thermal integration remains considerably less explored than RGB–LiDAR fusion. Challenges related to limited thermal texture, lower spatial resolution, multimodal correspondence extraction, and calibration reliability continue to motivate the development of dedicated thermal–LiDAR fusion frameworks operating under real measurement conditions.

### 2.3. 3D Thermal Mapping

Three-dimensional mapping of thermal information is an important research direction in the context of environmental monitoring, infrastructure diagnostics, and building energy analysis. Integration of spatial and thermal data provides identification of heat losses, detection of structural damage, and analysis of material physical properties [12].

Various methods for mapping thermal data into 3D space have been proposed in the literature, including approaches based on multi-view reconstruction, RGB-D systems, and integration of data from active distance sensors [35,36]. In many cases, filtering and interpolation techniques are applied to reduce the influence of measurement noise and improve temperature mapping accuracy [37].

Such solutions are used, among others, in building energy efficiency analysis, monitoring of industrial installations, and decision support systems for infrastructure management [8].

Despite progress in this area, there is still a lack of studies focusing on practical implementation of systems enabling precise assignment of thermal information to LiDAR point clouds using real measurement data.

## 3. System Overview

In the conducted experiments, a measurement setup consisting of an Ouster OS1 LiDAR scanner and a FLIR A70 thermal camera was used. The sensors were mounted on a common mechanical platform ensuring a stable and fixed relative position of both devices, which supports multi-sensor calibration and the integration of spatial and thermal data. Such a configuration allows simultaneous acquisition of information about the scene geometry and the temperature distribution of observed objects, which is essential for three-dimensional environmental analysis.

### 3.1. Hardware Setup

The Ouster OS1 LiDAR belongs to the class of sensors utilizing the Time-of-Flight (ToF) laser pulse measurement technology. The sensor generates a three-dimensional point cloud representing the geometric structure of the surrounding environment. Each point in the cloud is described by spatial coordinates (x,y,z) along with additional parameters such as the laser beam return intensity. The Ouster scanner is characterised by high spatial resolution and real-time operation capability, enabling accurate reconstruction of scene geometry in both indoor and outdoor environments. The sensor uses multi-beam laser emission, enabling spatial data acquisition over a wide field of view.

The main technical specifications of the LiDAR sensor are listed below:

- Number of measurement channels: 32/64 (depending on configuration);
- Measurement range: up to 120 m;
- Distance measurement accuracy: up to ±3 cm;
- Field of view (FoV): 360° (horizontal) × 45° (vertical);
- Scanning frequency: up to 10–20 Hz;
- Number of points per second: up to approx. 1,300,000 points/s;
- Communication interface: Ethernet;
- Data format: 3D point cloud (XYZ + intensity).

LiDAR data are used in the subsequent processing stage as a reference representation of the environment geometry, onto which temperature information obtained from the thermal camera is mapped.

The second sensor used in the experiments was the FLIR A70 thermal camera, enabling measurement of temperature distribution on the surfaces of observed objects. The camera operates in the Long-Wave Infrared (LWIR) range, allowing detection of thermal radiation independently of lighting conditions. The FLIR A70 generates a thermal image in which each pixel corresponds to a specific temperature value. The data are stored as radiometric images, enabling direct use of temperature values in further processing. The camera enables the identification of phenomena such as heat loss, non-uniform temperature distribution, and thermal anomalies.

The main parameters of the FLIR A70 camera are as follows:

- Detector resolution: 640×480 pixels;
- Spectral range: 7.5–14μm;
- Thermal sensitivity (NETD): <50 mK;
- Temperature measurement range: −20 °C to 550 °C;
- Frame rate: 30 Hz;
- Temperature accuracy: ±2 °C or ±2%;
- Communication interface: GigE Vision;
- Data format: radiometric temperature images.

Thanks to its ability to record precise temperature values, the thermal camera extends the geometric information obtained from the LiDAR with an additional physical dimension that describes the thermal properties of observed objects.

### 3.2. Measurement Setup and Data Acquisition

The LiDAR sensor and the thermal camera were mounted on a common mechanical structure ensuring stability of the measurement system and a fixed relative position between the sensors. Stable mounting of the sensors is crucial for the multi-sensor calibration process, as it supports estimation of a constant transformation between the coordinate systems of both devices.

**Figure 1:**
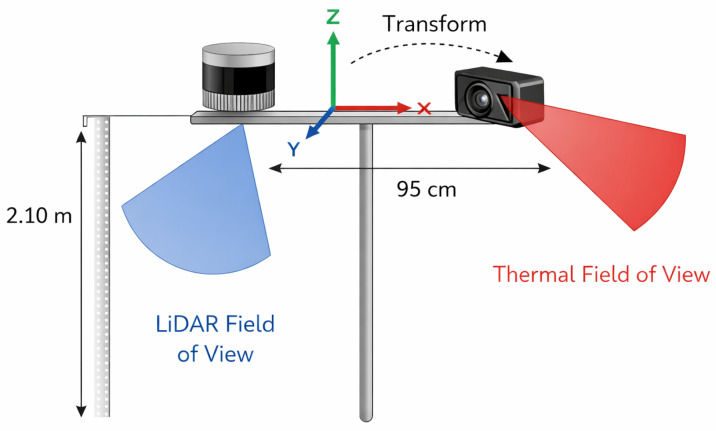
Configuration of the measurement system consisting of the Ouster OS1 LiDAR sensor and the FLIR A70 thermal camera mounted on a common horizontal beam. Both sensors are oriented in the same direction towards the observed scene, ensuring overlapping fields of view. The sensors are separated by approximately 95 cm and mounted on a support structure at a height of approximately 2.10 m above the ground. The figure also illustrates the coordinate system and the transformation between the LiDAR and thermal camera reference frames used in the multisensor calibration process.

The sensors were installed on a shared mounting beam attached to a vertical tripod with a height of approximately 2.1 m. Both devices were oriented in the same observation direction toward the scene. The distance between the centers of the optical coordinate systems of the sensors was approximately 95 cm. Such a configuration facilitates simultaneous observation of the same spatial region while maintaining a partially overlapping field of view of both sensors Figure 1.

The measurement setup was designed to enable simultaneous data acquisition from both sensors and their temporal synchronisation. Spatial data were obtained from the Ouster OS1 LiDAR scanner in the form of a 3D point cloud containing spatial coordinates of points and information about the laser beam return intensity. At the same time, the FLIR A70 thermal camera recorded temperature images in the form of radiometric thermal maps, in which each pixel corresponds to a temperature value.

The data acquisition process was performed sequentially, enabling timestamps to be assigned to data from both sensors. In each measurement step, a LiDAR point cloud and the corresponding thermal image were recorded. Data synchronisation was achieved by storing the acquisition time for both data types, enabling matching corresponding measurements in subsequent processing stages.

LiDAR data were stored as CSV files containing point coordinates (x,y,z) along with additional measurement parameters. Images from the thermal camera were stored as bitmap maps containing temperature values for each image pixel. This data format facilitates further processing using analytical tools and software libraries available in the Python environment.

The acquired data serve as the basis for subsequent multi-sensor calibration and the integration of spatial and thermal information. In the next processing stage, data from both sensors were transformed into a common coordinate system using the estimated geometric transformation between sensor reference frames. This enabled assigning temperature values to individual LiDAR points, extending the geometric representation of the scene with additional physical information.

The developed data processing pipeline consists of the following steps:

1. Acquisition of the LiDAR point cloud;
2. Acquisition of the thermal image;
3. Saving data together with timestamps;
4. Transformation of LiDAR points to the camera coordinate system;
5. Projection of 3D points onto the thermal image plane;
6. Assignment of temperature values to spatial points;
7. Estimation of the spatial coordinates of the hottest point.

The prepared data enable further spatial analysis of the scene enriched with thermal information, forming the basis for applications such as environmental monitoring, technical diagnostics, and energy performance analysis of objects.

## 4. Multi-Sensor Calibration

Multi-sensor calibration is a key stage in integrating data from a LiDAR scanner and a thermal camera. Its objective is to determine the geometric relationships between the coordinate systems of both sensors, enabling mutual mapping of spatial and thermal data. In practice, this process consists of two main stages: intrinsic calibration of the thermal camera and extrinsic calibration between the camera and the LiDAR scanner. The first stage estimates the camera model parameters that map spatial points onto the image plane, while the second determines the relative position and orientation of the sensors.

In the case of integration of a thermal camera and a LiDAR scanner, the calibration process is more challenging than in standard LiDAR–RGB systems. This results primarily from the lower spatial resolution of thermal images, limited contrast, and difficulties related to unambiguous detection of common features in both sensing modalities. Therefore, the appropriate selection of the calibration object, feature extraction procedure, and optimisation method for transformation parameters is of particular importance.

**Figure 2:**
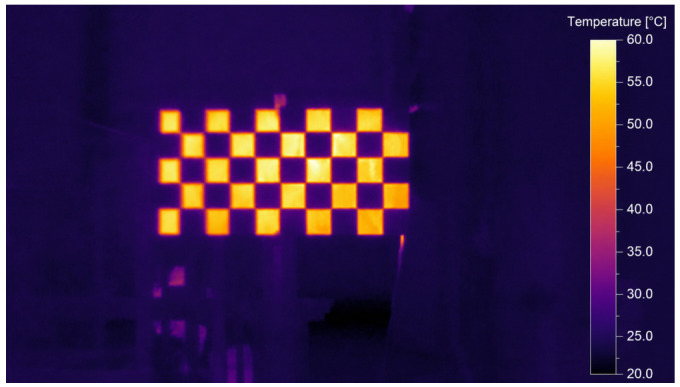
Calibration target consisting of a heated plate and non-heated cubic elements (10×10×6 cm), providing detectable features in both LiDAR and thermal data.

A specially prepared calibration target was used, consisting of an array of thermal points that served as simultaneous 3D spatial features (Figure 2). Cubes with dimensions 10×10×6 cm were mounted on a heating plate, where the cubes themselves did not heat up. This configuration produced spatial reference points together with heated regions. Due to their higher temperature relative to the background, these points were clearly visible in thermal camera images, while their spatial positions enabled accurate detection by the LiDAR scanner. An advantage of this solution is the ability to perform calibration in the absence of visible light and without optical markers.

### 4.1. Intrinsic Calibration of the Thermal Camera

Intrinsic calibration of the thermal camera aims to estimate the parameters of the projection model that describes the relationship between spatial points and their projections onto the image plane. In this work, the classical pinhole camera model was adopted, which is commonly used in calibration and image projection tasks. In this model, a spatial point expressed in the camera coordinate system is projected onto the image plane using the intrinsic parameter matrix.

The intrinsic parameter matrix can be expressed as:

$$K=\left[\begin{array}{ccc} f_{x} & 0 & c_{x}\\ 0 & f_{y} & c_{y}\\ 0 & 0 & 1 \end{array}\right],$$

where $f_{x}$ and $f_{y}$ denote the effective focal lengths in the horizontal and vertical directions, while $c_{x}$ and $c_{y}$ represent the coordinates of the principal point of the image. Depending on the required model accuracy, lens distortion parameters describing radial and tangential distortions were also considered.

Calibration of the thermal camera was performed using a series of calibration target images acquired from different positions and orientations. Compared to classical RGB camera calibration, this process involves additional challenges related to the characteristics of thermal images. In particular, the low contrast of the calibration pattern and the limited number of clearly visible feature points make accurate detection more difficult. For this reason, a calibration object providing a strong thermal response was used, ensuring that its features could be reliably detected in the thermal image.

### 4.2. Extrinsic Calibration (LiDAR–Thermal)

**Figure 3:**
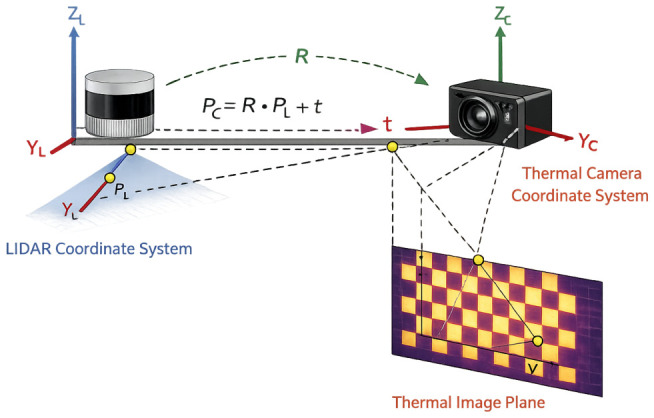
Diagram of extrinsic calibration between the LiDAR and the thermal camera. A 3D point $P_{L}$ from the LiDAR coordinate system is transformed into the camera coordinate system using the rotation matrix *R* and the translation vector *t*, and then projected onto the thermal image plane.

Extrinsic calibration aims to determine the rigid transformation between the LiDAR scanner’s and the thermal camera’s coordinate systems. This transformation facilitates mapping spatial points onto the thermal image and assigning temperature values to points of the 3D point cloud. The geometric relationship between the coordinate systems is illustrated in Figure 3.

The transformation between coordinate systems is described by the equation:

$$P_{c}=RP_{l}+t$$

where:

$P_{l}=\left(X_{l},Y_{l},Z_{l}\right)$ is a point in the LiDAR coordinate system,

$P_{c}=\left(X_{c},Y_{c},Z_{c}\right)$ is a point in the camera coordinate system,

R is the rotation matrix,

t is the translation vector.

The transformation parameters were estimated using a set of reference points observed simultaneously by both sensors. For this purpose, a calibration object visible both in the LiDAR point cloud and in the thermal image was used. Characteristic feature points of the calibration target were selected manually, which enabled obtaining a set of corresponding 2D–3D point pairs.

Calibration correspondences were established by selecting characteristic corner points of the dedicated calibration target that were clearly distinguishable in both the thermal image and the LiDAR point cloud. Particular attention was paid to selecting geometrically well-defined features with high thermal contrast to minimise ambiguity during correspondence assignment. The same physical target points were identified in both sensing modalities and subsequently used for Perspective-n-Point optimisation.

To reduce correspondence selection uncertainty, calibration points were distributed across the entire target surface rather than concentrated in a single region. This strategy improves geometric conditioning of the calibration problem and reduces the influence of individual point selection errors on the estimated transformation parameters.

No dedicated outlier rejection procedure was applied because all calibration correspondences were manually verified prior to optimisation. Nevertheless, the obtained reprojection errors indicate that no significant mismatches were present in the selected correspondence set.

Although manual correspondence selection introduces a degree of operator dependency, it was adopted in this study due to the limited number of clearly distinguishable features simultaneously visible in both LiDAR and thermal modalities. The use of a dedicated thermally distinguishable calibration target reduces ambiguity during correspondence identification and improves consistency of point selection. Nevertheless, small variations in manually selected image coordinates may influence the estimated transformation parameters and contribute to residual reprojection error.

In the first stage, thermal data stored as a matrix of temperature values was converted into an image with a resolution of 640×480 pixels. Next, the coordinates of characteristic points on the thermal image were determined in pixel form (u,v). Corresponding spatial points were selected in the LiDAR point cloud as coordinates (X,Y,Z). As a result, a set of reference points enabling estimation of the geometric transformation was obtained.

The Perspective-n-Point (PnP) algorithm was used to estimate the transformation parameters. This method allows estimation of the camera pose relative to a known set of spatial points. Rotation and translation parameters were obtained by minimizing the reprojection error between observed image points and points obtained after projection of spatial points.

The transformation matrix can be written as:

$$T=\left[\begin{array}{cc} R & t\\ 0 & 1 \end{array}\right]$$

After estimating the transformation, a point in the camera coordinate system can be projected onto the image plane according to:

$$u=f_{x}\frac{X_{c}}{Z_{c}}+c_{x}$$

$$v=f_{y}\frac{Y_{c}}{Z_{c}}+c_{y}$$

where (u,v) denote the pixel coordinates in the thermal image.

As shown in Figure 3, the estimated transformation facilitates unambiguous assignment of temperature values to spatial points of the LiDAR point cloud, which forms the basis for further spatial–thermal analysis of the environment.

### 4.3. Calibration Accuracy

The accuracy of the multi-sensor calibration was evaluated using reprojection error, which measures the difference between the actual position of a point in the thermal image and the position obtained by projecting it using the estimated geometric transformation. The reprojection error is one of the most commonly used metrics for assessing the quality of camera-LiDAR calibration, as it directly reflects the accuracy of mapping spatial data into the image coordinate system.

The reprojection error for a single point was calculated according to:

$$e_{i}=\sqrt{{\left(u_{i}-{\hat{u}}_{i}\right)}^{2}+{\left(v_{i}-{\hat{v}}_{i}\right)}^{2}}$$

where:

$\left(u_{i},v_{i}\right)$—actual coordinates of the point in the thermal image,

$\left({\hat{u}}_{i},{\hat{v}}_{i}\right)$—coordinates obtained after projection of the LiDAR point using the estimated transformation.

The mean reprojection error expressed as the Root Mean Square Error (RMSE) was calculated according to:

$$RMSE=\sqrt{\frac{1}{N}\sum_{i=1}^{N}e_{i}^{2}}$$

where *N* denotes the number of points used in the calibration process.

In the conducted experiments, 6 to 12 characteristic points of the calibration target were used, visible in both the LiDAR point cloud and the thermal image. The points were distributed across the camera’s field of view to improve the stability of transformation parameter estimation.

The obtained reprojection error values are presented in Table 2. As the number of reference points increases, the error decreases, confirming the correctness of the adopted transformation estimation method. For 12 points, an RMSE of less than 1 pixel was achieved, indicating very good agreement between the LiDAR data and the thermal image.

Considering the resolution of the thermal camera of 640×480 pixels, the obtained reprojection error can be considered small and sufficient for the correct assignment of temperature values to 3D point cloud data. Small shifts of 1–2 pixels have a limited impact on temperature-mapping accuracy, particularly for objects with relatively uniform temperature distributions.

The calibration accuracy is primarily influenced by:

- The number of reference points;
- Their spatial distribution;
- The quality of point detection in the thermal image;
- The accuracy of point selection in the LiDAR point cloud.

In particular, it is important to use points distributed across different regions of the image, which reduces uncertainty in the estimation of transformation parameters.

The obtained results indicate that the proposed calibration method provides an accurate determination of the geometric relationship between the sensors, forming the basis for further integration of spatial and thermal data.

### 4.4. Sensitivity Analysis of Calibration Accuracy

To evaluate the calibration procedure’s sensitivity to the number of reference correspondences, the calibration was repeated with different subsets of calibration points. Table 1 demonstrates a clear reduction in reprojection error as the number of calibration points increases.

The largest improvement was observed when increasing the number of calibration points from six to ten, where the RMSE decreased from 2.1 px to 1.2 px. Further increasing the number of points to twelve reduced the error only marginally to 0.9 px. This behaviour suggests that the Perspective-n-Point optimization becomes increasingly stable as additional geometric constraints are introduced and gradually reaches a saturation region in which further correspondences provide only limited improvement.

The results indicate that calibration accuracy is sensitive to the number and distribution of calibration points, particularly when only a few correspondences are available. At the same time, the relatively small improvement observed beyond ten points suggests that the estimated transformation becomes stable once a sufficient number of well-distributed reference features is provided.

The results also indicate that not only the number but also the spatial distribution of calibration points affects the stability of the estimated transformation. Correspondences distributed across the entire calibration target provide stronger geometric constraints and reduce sensitivity to local selection inaccuracies.

## 5. Thermal Mapping onto LiDAR Point Cloud

After determining the intrinsic calibration parameters of the camera and the extrinsic transformation between the LiDAR and thermal camera coordinate systems, it becomes possible to assign thermal information to spatial points in the LiDAR point cloud. This process consists of transforming 3D points into the camera coordinate system, projecting them onto the thermal image plane, and then retrieving the corresponding temperature values. As a result, an extended point cloud is obtained in which each spatial point is associated with an additional attribute describing temperature. Such a data representation serves as the basis for further spatial–thermal analysis of the scene, including the localisation of regions with elevated temperatures and the estimation of the spatial coordinates of the hottest point.

### 5.1. Coordinate Transformation and Point Projection

The first stage of thermal data mapping is transforming LiDAR point cloud coordinates into the thermal camera’s coordinate system. For each point $P_{l}=\left(X_{l},Y_{l},Z_{l}\right)$, the previously estimated transformation matrix $T_{\mathrm{LiDAR}\to \mathrm{FLIR}}$ is applied, which contains both rotational and translational components. In the implementation, each point is represented in homogeneous coordinates and multiplied by the transformation matrix in order to obtain its coordinates in the camera reference frame. This procedure was implemented both in the calibration stage and in the temperature-assignment module.

After transforming a point into the camera coordinate system, it is projected onto the image plane using the classical perspective camera model and the intrinsic parameter matrix of the FLIR A70 camera. For a point $P_{c}=\left(X_{c},Y_{c},Z_{c}\right)$, the image coordinates are determined according to:

$$u=\frac{f_{x}X_{c}}{Z_{c}}+c_{x}$$

$$v=\frac{f_{y}Y_{c}}{Z_{c}}+c_{y}$$

In practice, this means that each 3D point in the LiDAR point cloud can be assigned to a single pixel in the thermal image, provided the point lies in front of the camera and its projection falls within the image boundaries. The projection procedure is performed only after applying the transformation matrix and the known camera projection parameters.

An important element of the algorithm is the visibility filter. In the implementation, all points with $Z_{c}\leq 0$ are rejected, as they lie behind the camera and cannot be projected onto the image plane. Additionally, it is verified whether the computed coordinates (u,v) lie within the thermal image resolution range, i.e.,

0≤u<W,0≤v<H

If this condition is not satisfied, the point is considered not visible from the camera perspective and no temperature value is assigned. This filtering reduces incorrect associations between spatial points and pixels located outside the camera field of view.

### 5.2. Temperature Assignment to 3D Points

After determining the image coordinates for visible points, the actual temperature assignment is performed. In the basic version of the algorithm, the temperature of a 3D point is directly obtained from the thermal image as the pixel value:

T=thermal[v,u]

For each successfully projected point, the corresponding temperature value is stored, while for non-visible points the value NaN is assigned. As a result, a temperature vector is generated for the LiDAR point cloud. The data are then merged and stored as an extended point cloud in the format:

(X,Y,Z,Temperature)

In a more advanced implementation, handling of missing thermal data is also considered. If direct temperature reading from the pixel returns a zero or undefined value, bilinear interpolation is applied in order to estimate temperature based on neighbouring pixel values. If this step does not provide a valid result, an additional 3×3 neighbourhood window is used, within which the mean value of valid temperatures is computed. This approach reduces the influence of missing data, quantisation errors, and isolated outlier pixels.

Although the basic experiments used direct temperature assignment based on a single pixel, the interpolation and neighbourhood analysis approach constitutes a valuable extension of the algorithm and improves its numerical robustness.

After assigning temperature values to all valid points, the point with the highest temperature is determined. In the implementation, this is achieved by searching for the maximum value among points with valid temperature assignments. The spatial coordinates of this point in the LiDAR coordinate system are then stored, its distance from the sensor is computed, and optionally, its coordinates can be transformed relative to another device using a known geometric offset.

### 5.3. Implementation Details

The complete data integration algorithm was implemented in Python using the NumPy, OpenCV, Matplotlib, and Open3D libraries. NumPy was used for matrix operations, point cloud storage, and numerical computations. OpenCV was applied both during calibration and projection stages, particularly for estimating the transformation using the solvePnP method and for processing thermal images. Matplotlib was used for visualisation and saving normalised thermal images, while Open3D was used to handle the point cloud, select reference points, and visualise the resulting point cloud with assigned temperature values.

From the implementation perspective, the algorithm can be represented as the following sequence of operations:

1. loading the LiDAR point cloud;
2. loading the thermal image and transformation matrix;
3. Transforming points to the camera coordinate system;
4. Projection onto the image plane;
5. Assigning temperature values;
6. Saving the extended point cloud to the output file.

The final stage involves 3D visualisation, in which point colours are scaled by temperature using the inferno colourmap, and the point with the maximum temperature is marked. Example visualisations confirm that the obtained representation allows the correct localisation of the hottest regions in the Algorithm 1.

The algorithm can be summarised in the following pseudocode:
**Algorithm 1** Thermal mapping onto LiDAR point cloud**Require:** point cloud *P*, thermal image *I*, intrinsic matrix *K*, transformation matrix *T* **Ensure:** augmented point cloud $P_{T}=\left\{\left(X,Y,Z,T\right)\right\}$
  1:**for** each point p∈P **do**  2:      transform *p* to camera coordinate system using *T*  3:      **if** $Z_{c}>0$ **then**  4:            project point onto image plane using *K*  5:            **if** 0≤u<W and 0≤v<H **then**  6:                  read temperature value from image I(u,v)  7:            **else**  8:                  assign temperature NaN  9:            **end if**10:      **else**11:            assign temperature NaN12:      **end if**13:**end for**14:find point with maximum temperature15:save augmented point cloud $P_{T}$


## 6. Experimental Results

The proposed LiDAR–thermal data fusion method was evaluated using real measurements acquired with the experimental setup described in Section 3. The objective of the experiments was to verify whether the estimated calibration parameters and the implemented mapping procedure enable reliable transfer of temperature information from the thermal image to the LiDAR point cloud. The evaluation included both qualitative analysis of the obtained 3D thermal representations and a quantitative assessment of mapping accuracy.

### 6.1. Experimental Setup

The experiments were carried out in an indoor laboratory environment with structural elements, furniture, and a thermal calibration target, all within the common field of view of both sensors. The calibration target produced a clearly distinguishable temperature pattern in the thermal image and at the same time remained detectable in the LiDAR point cloud, which made it possible to verify both geometric alignment and thermal assignment.

A series of synchronized LiDAR point clouds and thermal images was acquired for the analysed scene. The measurements were performed under stable indoor conditions, with minimal airflow and no rapid ambient temperature changes, thereby reducing the influence of external factors on the thermal readings. The collected dataset included raw LiDAR point clouds, radiometric thermal data, and the corresponding processed outputs used for calibration and thermal mapping.

The evaluation focused on three aspects: the visual consistency of the thermal projection in the 3D scene, the effect of calibration quality on the final mapping result, and the system’s ability to identify regions of elevated temperature in spatial coordinates.

### 6.2. Qualitative Results

**Figure 4:**
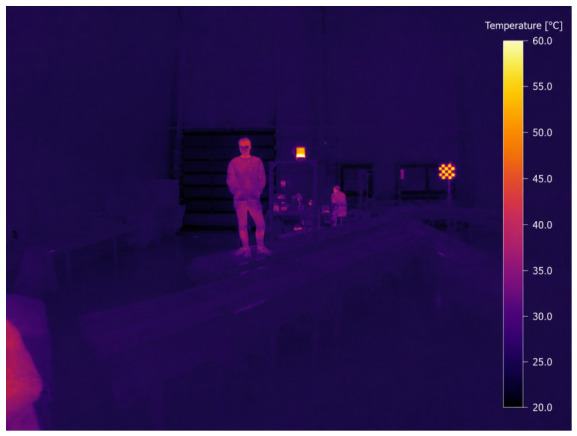
Example thermal image acquired during the experiment. The calibration target is visible as a high-contrast temperature pattern.

Figure 4 presents an example of a thermal image acquired during the experiment. The calibration target is clearly visible as a high-contrast region, which confirms that the selected pattern can be reliably identified in the thermal modality.

**Figure 5:**
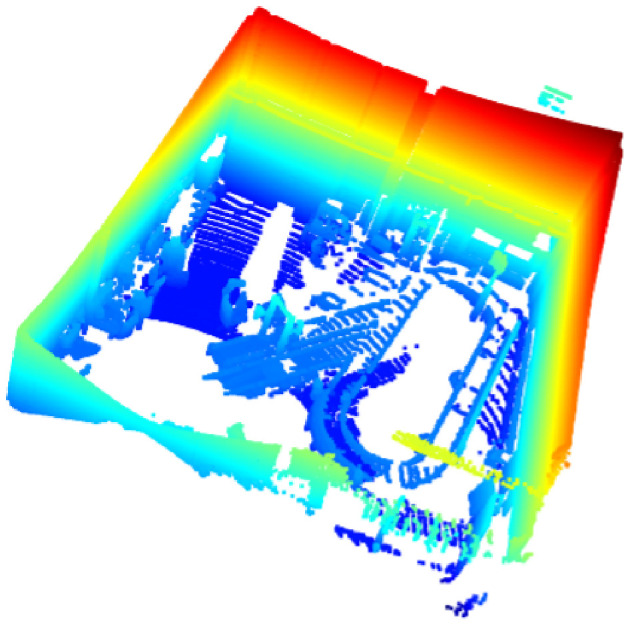
Raw LiDAR point cloud representing the observed scene before thermal information assignment.

Figure 5 shows the raw LiDAR point cloud representing the scene geometry prior to temperature assignment. The point cloud contains only spatial information and provides no indication of thermal characteristics.

**Figure 6:**
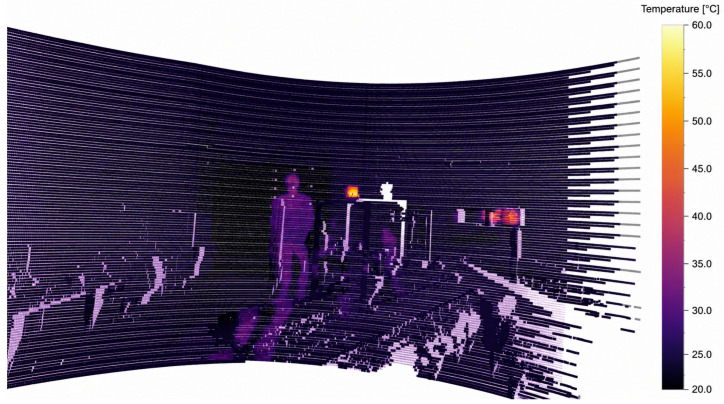
LiDAR point cloud augmented with thermal information. Point colour corresponds to the assigned temperature value.

After applying the estimated transformation and projecting the LiDAR points onto the thermal image plane, temperature values were assigned to the visible 3D points. The resulting thermally augmented point cloud is presented in Figure 6. It can be observed that the elevated-temperature region is spatially aligned with the thermal target, confirming the correctness of the geometric mapping.

An additional functionality of the proposed method is the automatic identification of the spatial location corresponding to the highest observed temperature. After assigning temperature values to all visible points in the LiDAR point cloud, the algorithm determines the point with the global maximum temperature. The coordinates of this point are then returned in the LiDAR reference coordinate system, enabling direct spatial localisation of the hottest region in the observed scene.

The estimated spatial coordinates of the hottest point were validated using independent distance measurements. A laser distance meter was used to measure the distance between the measurement system and the thermal target. The measured distance was then compared with the distance calculated from the LiDAR point coordinates. This comparison allowed estimation of the localisation error of the hottest point relative to the measurement setup.

The results confirmed that the proposed method allows correct identification of the spatial position of the highest temperature region. The estimated position of the hottest point remained consistent with the actual position of the heated calibration object, demonstrating that the combined calibration and projection procedure preserves spatial consistency between the thermal and LiDAR data domains.

### 6.3. Quantitative Evaluation

The quantitative evaluation was based on the consistency of the temperature mapping and on the calibration accuracy discussed in Section 4. In practical terms, the quality of thermal assignment depends directly on reprojection accuracy, since spatial misalignment between the thermal image and the point cloud can lead to incorrect temperature assignments to 3D points.

Table 3 summarizes the relation between the number of calibration points and the obtained reprojection error. As expected, increasing the number of reference points improves the estimated transformation and reduces the mapping uncertainty.

In addition to reprojection accuracy, the spatial accuracy of the hottest-point localisation was evaluated. The coordinates of the point corresponding to the maximum temperature value were compared with reference distance measurements obtained using a laser distance meter. The comparison was performed relative to the measurement system coordinate frame.

The results confirm that the proposed method effectively fuses LiDAR geometry and thermal measurements, providing a coherent three-dimensional representation of the observed scene enriched with temperature information. The achieved localisation accuracy of the hottest point is consistent with the LiDAR sensor’s nominal accuracy and confirms the correctness of the transformation parameters estimated during calibration.

The experimental evaluation indicates that the calibration procedure plays a critical role in the final accuracy of thermal mapping. A series of tests performed with different numbers and spatial configurations of calibration points showed that the most stable results are obtained when 6–12 well-distributed calibration correspondences are used. Within the investigated range (6–12 points), the improvement becomes marginal beyond ten correspondences. Once a sufficient number of constraints is reached, the optimisation problem becomes well-conditioned, and additional points may introduce redundancy and increase sensitivity to localisation errors in individual correspondences.

The spatial distribution of calibration points also significantly influences mapping accuracy. The best results were obtained when calibration points were located on a common plane visible in both sensing modalities. In this configuration, the estimated transformation provides consistent geometric alignment between the LiDAR point cloud and the thermal image. When calibration points are distributed irregularly in space and do not form a coherent planar structure, the reprojection error increases and geometric distortions may appear during thermal projection onto the point cloud.

Another important observation is that the highest localisation accuracy is achieved when the analysed thermal object is located close to the calibration plane. Since the extrinsic parameters are estimated based on features lying on a specific surface, the transformation model is most accurate in its neighbourhood. For objects located far outside the calibration plane, the accumulated geometric error may increase, reducing the accuracy of the estimated spatial position of the hottest point.

The experimental results (Table 4) also show that the presence of additional objects behind the analysed thermal target may affect the accuracy of temperature assignment. Thermal cameras register surface radiation integrated along the line of sight, while LiDAR measures geometric distance to the first detected surface. When multiple objects overlap in the camera view, the thermal sensor’s temperature readings may correspond to background structures rather than the intended target. This effect may lead to incorrect association of thermal values with LiDAR points and increased localisation error.

Additional limitations are related to the spatial resolution of the thermal camera and partial overlap of the sensor fields of view. Points located outside the thermal image area or occluded from the camera perspective cannot be assigned reliable temperature values. Furthermore, local inaccuracies may occur near temperature discontinuities, where even small reprojection errors may result in assignment of neighbouring pixel values. This effect is particularly visible along object edges and in regions characterised by strong thermal gradients.

Despite these limitations, the experimental study confirms that the proposed approach enables reliable estimation of the spatial coordinates of thermal anomalies in three dimensions. The obtained accuracy, with mean localisation error on the order of a few centimetres at a distance of approximately 12.3 m, demonstrates the practical applicability of the proposed method in inspection, monitoring, and diagnostic tasks requiring integration of geometric and thermal information.

A statistical analysis of the localization error was additionally performed using the twenty independent measurements presented in Table 5. The obtained results indicate a mean localization error of 4.1 cm and a median error of 4.0 cm, demonstrating good consistency between repeated measurements. The observed localization error ranged from 0.4 cm to 7.3 cm, with a standard deviation of 1.9 cm.

The calculated 95% confidence interval of 3.2–5.0 cm indicates that the localization accuracy remains stable across repeated measurements and confirms the reliability of the proposed thermal anomaly localization framework under the tested experimental conditions.

The relatively small spread of localization errors suggests that the estimated sensor transformation remains stable after calibration and that the projection-based thermal mapping procedure is not excessively sensitive to small variations in individual measurements. Nevertheless, the presented results correspond to controlled indoor conditions, and additional experiments conducted in dynamic or outdoor environments will be necessary to fully characterize the robustness of the proposed framework.

## 7. Discussion

The obtained experimental results confirm that the proposed framework enables effective integration of spatial LiDAR measurements and temperature information acquired from a thermal camera. The developed processing pipeline allows assigning temperature values to three-dimensional point cloud data and determining the spatial coordinates of thermal anomalies. Experimental validation demonstrated mean hottest-point localisation errors of a few centimetres at approximately 12.3 m, indicating that accurate thermal localisation can be achieved using a geometry-based multimodal sensing approach.

The statistical analysis of localisation accuracy further confirmed the stability of the proposed framework. The relatively small difference between the mean and median localization errors, together with the obtained confidence interval, indicates consistent performance across repeated measurements. These results suggest that the proposed thermal mapping procedure provides reliable localization under the investigated experimental conditions.

An important observation from the experimental evaluation is that localization accuracy depends more strongly on calibration quality than on the thermal mapping procedure itself. The sensitivity analysis demonstrated a systematic reduction of reprojection error with increasing numbers of calibration correspondences. Improved calibration accuracy directly translated into better spatial consistency between thermal measurements and LiDAR geometry, indicating that precise estimation of the extrinsic transformation remains the dominant factor affecting final localization performance. Consequently, future improvements should focus primarily on correspondence extraction, calibration automation, and uncertainty reduction rather than modifications of the projection procedure itself.

Although the experimental validation was conducted at a distance of approximately 12.3 m, the obtained localisation error is largely determined by calibration accuracy rather than distance itself. Assuming a constant reprojection error below 1 pixel and comparable thermal target visibility, similar centimetre-level localisation accuracy is expected at shorter distances (e.g., 5–8 m), where the spatial footprint corresponding to a single thermal pixel is smaller. Nevertheless, dedicated experiments at multiple distances are required to quantitatively verify this assumption and characterise distance-dependent error propagation.

Compared with many recent LiDAR–camera and LiDAR–thermal calibration approaches reported in the literature [18,31,32], the proposed framework focuses specifically on integration of radiometric thermal imagery and spatial LiDAR measurements for thermal anomaly localization. Thermal imaging introduces additional challenges due to lower spatial resolution, reduced texture information, and limited availability of distinctive multimodal features. While recent deep-learning-based methods [32,33] offer greater automation and robustness, they typically require large annotated datasets, substantial computational resources, and extensive training. In contrast, the proposed framework relies on a geometry-based calibration strategy that remains interpretable, computationally efficient, and suitable for deployment in practical engineering applications.

As shown in Table 6, most existing studies focus primarily on sensor calibration or multimodal data alignment, whereas the proposed framework additionally enables direct three-dimensional localisation of thermal anomalies using real radiometric thermal data.

Nevertheless, geometry-based approaches may become less effective in environments characterised by sparse thermal features, severe sensor noise, partial target visibility, or challenging environmental conditions. In such cases, learning-based methods may potentially provide improved robustness through data-driven feature extraction and correspondence estimation. Therefore, the selection of an appropriate fusion strategy should depend on the intended application, available computational resources, and availability of representative training data.

Although the proposed framework is primarily engineering-oriented rather than focused on introducing a fundamentally new calibration model, its contribution lies in the practical integration of thermal and spatial sensing modalities under real measurement conditions. The proposed thermally distinguishable calibration target and complete end-to-end processing pipeline provide a reproducible solution for three-dimensional thermal anomaly localization using commercially available sensing hardware.

Several limitations of the proposed framework should be acknowledged. First, the extrinsic calibration procedure relies on manually selected multimodal correspondences. Although the dedicated calibration target reduces ambiguity during correspondence identification, manual point selection may introduce operator-dependent variability and limit scalability in large-scale deployments. This limitation becomes particularly important when only a small number of calibration correspondences is available, as even minor selection inaccuracies may propagate into the estimated transformation parameters and affect final thermal localisation accuracy.

Second, the experimental validation was conducted in a controlled indoor environment using a static scene. Consequently, the reported localisation accuracy should be interpreted as performance achieved under laboratory conditions rather than as a direct indicator of accuracy in arbitrary real-world environments.

Furthermore, differences in spatial resolution between the LiDAR sensor and the thermal camera may affect temperature assignment accuracy, particularly near object boundaries and regions characterised by strong thermal gradients. In such situations, even small reprojection inaccuracies may result in the assignment of neighbouring temperature values, reducing thermal localisation precision.

Temporal synchronisation also remains an important practical consideration. In dynamic scenes, even small timing offsets between LiDAR acquisition and thermal image capture can lead to incorrect association of temperature values with spatial points. Similarly, occlusion effects may lead to incorrect temperature assignment when multiple objects overlap within the thermal camera field of view. Although these phenomena had a limited influence in the presented experiments, they may become more significant in complex real-world environments.

Despite these limitations, the presented results demonstrate that accurate thermal anomaly localisation can be achieved using a geometry-based fusion framework. Additional validation under diverse environmental conditions and operating scenarios will further strengthen the generalisability of the proposed approach.

Despite the identified limitations, the proposed method demonstrates strong potential for application in building energy diagnostics, industrial inspection, technical infrastructure monitoring, mobile robotics, and autonomous systems. The ability to determine the three-dimensional coordinates of thermal anomalies provides valuable information for inspection and monitoring tasks where accurate spatial localization of heat sources is required.

Future work will focus on automatic correspondence extraction, calibration automation, hardware-level sensor synchronisation, and evaluation under a wider range of environmental conditions. Additional research may also investigate the integration of machine-learning-based feature extraction techniques and higher-resolution thermal sensors to further improve localisation accuracy and robustness.

## 8. Conclusions

This paper presented a method for integration of LiDAR spatial data and thermal imagery enabling three-dimensional localisation of temperature anomalies in the observed environment. The proposed approach includes the development of a complete measurement setup, a multi-sensor calibration procedure, and an algorithm for the projection of thermal information onto a LiDAR point cloud. The obtained results confirm that it is possible to assign temperature values to 3D points with accuracy consistent with the nominal precision of the applied sensors.

Experimental evaluation demonstrated that the proposed calibration methodology enables stable estimation of the geometric relationship between the LiDAR and thermal camera coordinate systems. The obtained reprojection errors and localisation accuracy of the hottest point indicate that the method allows reliable spatial identification of thermal features at distances exceeding 12 m. The results also confirm that proper selection and spatial distribution of calibration points significantly influences the final mapping accuracy.

The practical value of the proposed solution lies in its applicability to tasks requiring precise localisation of heat sources in three-dimensional space. Potential application areas include building energy diagnostics, monitoring of industrial infrastructure, inspection of technical installations, robotics, and autonomous systems. The ability to combine geometric and thermal information in a unified spatial representation increases the interpretability of measurement data and enables more effective detection of thermal anomalies.

Future work will focus on extending the proposed method towards real-time processing, enabling continuous acquisition and integration of multimodal sensor data. Further research may include the use of machine learning methods for automatic detection of calibration features and improvement of correspondence estimation between sensing modalities. Additionally, experiments conducted in larger and more complex environments may allow evaluation of the scalability of the proposed approach and its robustness under more challenging measurement conditions.

The presented results confirm that integration of LiDAR and thermal data constitutes a promising direction in the development of multi-sensor perception systems, enabling a more comprehensive understanding of the observed environment and supporting advanced diagnostic and monitoring applications.

## Figures and Tables

**Table 1:** Comparison of the proposed framework with selected LiDAR–thermal and LiDAR–camera calibration approaches.

Method	Thermal	Real Data	Learning-Based	Main Characteristics
Park et al. [21]	No	Yes	No	Planar calibration target
Verma et al. [22]	No	Yes	No	Targetless calibration
Dalirani and El-Sakka [18]	Yes	Yes	No	Human-based correspondence matching
CalibRefine [24]	No	Yes	Yes	Deep-learning targetless calibration
Kim et al. [26]	Yes	Yes	Yes	Thermal–LiDAR fusion for perception
Proposed method	Yes	Yes	No	Thermal anomaly localization, LWIR calibration target, interpretable pipeline

**Table 2:** Reprojection error obtained for different numbers of calibration points.

Number of Points	RMSE [px]	Max Error [px]
6	2.1	3.8
8	1.6	2.9
10	1.2	2.1
12	0.9	1.8

**Table 3:** Influence of the number of calibration points on reprojection error and qualitative mapping quality.

Number of Points	RMSE [px]	Mapping Quality
6	2.1	acceptable
8	1.6	good
10	1.2	very good
12	0.9	high

**Table 4:** Localization accuracy of the hottest point for 20 measurements.

No.	*X* [m]	*Y* [m]	*Z* [m]	Dist_*est*_ [m]	Dist_*meas*_ [m]	Error [cm]
1	12.21	0.84	1.32	12.33	12.30	3.1
2	12.18	0.79	1.29	12.29	12.30	1.2
3	12.26	0.82	1.34	12.36	12.30	5.8
4	12.23	0.88	1.31	12.35	12.30	5.0
5	12.19	0.81	1.28	12.28	12.30	2.0
6	12.25	0.83	1.35	12.37	12.30	6.4
7	12.20	0.86	1.30	12.32	12.30	2.4
8	12.27	0.80	1.33	12.36	12.30	6.0
9	12.24	0.85	1.31	12.34	12.30	4.1
10	12.22	0.87	1.29	12.33	12.30	3.0
11	12.28	0.82	1.36	12.38	12.30	7.3
12	12.18	0.84	1.27	12.27	12.30	3.2
13	12.23	0.89	1.32	12.35	12.30	4.9
14	12.26	0.81	1.34	12.36	12.30	5.7
15	12.21	0.86	1.30	12.32	12.30	2.6
16	12.24	0.83	1.33	12.34	12.30	3.8
17	12.19	0.88	1.28	12.30	12.30	0.4
18	12.27	0.79	1.35	12.37	12.30	6.8
19	12.22	0.84	1.31	12.33	12.30	2.9
20	12.25	0.82	1.34	12.36	12.30	5.5
Mean	–	–	–	12.33	12.30	4.1
Std	–	–	–	0.03	0.00	1.9

**Table 5:** Statistical summary of hottest-point localization error.

Metric	Value [cm]
Mean error	4.1
Median error	4.0
Standard deviation	1.9
Minimum error	0.4
Maximum error	7.3
95% confidence interval	3.2–5.0

**Table 6:** Comparison of the proposed framework with selected LiDAR–thermal calibration and fusion approaches reported in the literature.

Method	Thermal Data	Calibration	3D Localisation
Dalirani and El-Sakka [18]	Yes	Yes	No
Niskanen et al. [30]	Yes	Partial	No
Zhang et al. [31]	Yes	Yes	No
CalibFormer [32]	No	Yes	No
Proposed method	Yes	Yes	Yes

## Data Availability

No new data were created or analyzed in this study.
